# High-performance Fe–Al@BTC MOF for supercapacitor and antibacterial applications: experimental, DFT, and molecular docking studies

**DOI:** 10.1038/s41598-026-43631-4

**Published:** 2026-04-02

**Authors:** Eman Abdelnasser, Abdulrhman M. Alaraj, Mahmoud Abdelfatah, Abdelhamid El-Shaer

**Affiliations:** 1 Chemistry Department, Faculty of Science, Kafrelsheikh University, El-Geish Street, P.O. Box 33516, Kafrelsheikh, Egypt; 2 Physics Department, Faculty of Science, Kafrelsheikh University, Kafrelsheikh, 33516 Egypt; 3 Nano Science and Technology Program, Faculty of Science, Kafrelsheikh University, Kafrelsheikh, 33516 Egypt

**Keywords:** Metal-organic frameworks (MOF), Photoelectrochemical properties, Supercapacitor, *Bacillus* bacterial cells, Docking investigation, Computational investigation, Chemistry, Materials science

## Abstract

A crystal, highly efficient, environmentally friendly, and low-cost metal-organic framework iron–aluminium-based metal–organic framework composed of Fe³⁺/Al³⁺ nodes coordinated with 1,3,5-benzenetricarboxylate (BTC) linkers (Fe–Al@BTC) was synthesized by the hydrothermal method. Photoelectrochemical properties of MOF were evaluated employing Mott-Schottky and EIS Measurements. Flat band potential and carrier density were 0.76 V and 1.3 × 10^20^ cm^− 3^. The measurements confirmed that Fe-Al@BTC is an n-type semiconductor. It exhibited promising electrochemical properties where charge transfer resistance and double-layer capacitance were observed at the electrode/electrolyte interface. Moreover, at a scan rate of 10 mV/s, the specific capacitance of Fe-Al@BTC MOF from cyclic voltammetry is 339.24 F/g. The structure BTC and MOF were optimized by DFT/ B3LYP 6-31G (d, p) to clarify their physical descriptor and identify their HOMO-LUMO band gap, which was more correlated with Physical and biological results. Furthermore, the antibacterial activity of Fe-Al @BTC was evaluated by optical density measurements and the cut plug method. It showed remarkable inhibition of bacterial growth by 100% at a concentration of 600 mg\L. Moreover, a molecular docking study of Fe-Al @BTC was performed to understand molecular interaction with *Bacillus subtilis* ATCC 6633 protein and its reactivity. Our results indicate that Fe-Al @BTC is a promising candidate for energy and environmental applications.

## Introduction

The development of multifunctional materials capable of simultaneously delivering efficient electrochemical energy storage and antibacterial activity remains a significant challenge; in this work, we introduce a bimetallic Fe–Al@BTC metal–organic framework that integrates pseudocapacitive charge storage^1^ and antibacterial functionality within a single MOF platform, demonstrating strong multifunctional performance for a supercapacitor^2,3^ and environmental applications^4^. Energy shortage has become one of the most critical global challenges due to rapid population growth, industrial expansion, and increasing technological demands. The growing consumption of fossil fuels and the associated environmental impact have intensified the need for sustainable and efficient energy storage solutions^5,6^.

Consequently, the development of advanced energy storage devices capable of delivering high power and energy density has attracted considerable scientific attention^7^. Among various energy storage systems, supercapacitors are regarded as promising candidates owing to their fast charge–discharge capability, high power density, long cycling stability, and environmentally friendly nature^8^. With the global energy demand expected to double by 2050, the advancement of efficient energy storage technologies has become increasingly essential^9,10^.

Alongside energy challenges, environmental pollution, particularly water contamination, poses a serious threat to ecosystems and public health. Environmental pollution includes chemical contaminants such as organic compounds, heavy metals, and radioactive materials, as well as biological pollutants such as pathogenic bacteria and antibiotic residues^11,12^. The increasing resistance of bacteria to conventional antibiotics has further aggravated this problem, leading to limited treatment options and higher healthcare costs^13,14^. As bacteria evolve rapidly, the effectiveness of traditional antimicrobial agents diminishes, necessitating the development of alternative and sustainable antibacterial strategies^15^. Therefore, multifunctional materials capable of addressing both environmental pollution and bacterial contamination are urgently required. Metal-organic frameworks have emerged as promising electrode materials for supercapacitors due to their tunable porosity, redox-active metal centers, and high surface area. Separately, MOFs have also demonstrated effective antibacterial properties through metal ion release, surface interactions, and defect-induced oxidative stress^16–25^. However, reports integrating both functionalities within a single MOF system remain limited. The Fe–Al@BTC MOF presented in this work bridges this gap by combining pseudocapacitive charge storage with antibacterial activity^26^.

Advanced functional materials have emerged as effective solutions to simultaneously tackle energy storage and environmental challenges. Among them, metal–organic frameworks (MOFs), composed of metal ions coordinated with organic ligands, have attracted significant attention due to their high surface area, tunable porosity, structural diversity, and chemical versatility. These unique characteristics make MOFs excellent candidates for electrochemical energy storage applications, particularly as electrode materials for supercapacitors, where ion diffusion and charge transfer play critical roles. In addition, MOFs have demonstrated remarkable antibacterial activity through mechanisms such as metal ion release, reactive oxygen species generation, and membrane disruption^15^.

In this study, a bimetallic iron–aluminum-based metal–organic framework coordinated with 1,3,5-benzenetricarboxylate (Fe-Al@BTC MOF) was synthesized and explored as a dual-functional material for energy storage and antibacterial applications. The structural, optical, electrochemical, and photoelectrochemical properties of the synthesized MOF were systematically investigated. Furthermore, its antibacterial activity against *Bacillus* bacterial cells was evaluated experimentally and supported by density functional theory (DFT) calculations and molecular docking studies to elucidate the structure–property–activity relationship. The results demonstrate that Fe–Al@BTC MOF is a promising candidate for high-performance supercapacitors and environmental applications.

## Materials and methods

### Chemicals

All chemicals used in this study were of analytical grade and used without further purification. Iron(III) nitrate nonahydrate (Fe(NO₃)₃·9 H₂O, ≥ 99%, CAS: 7782-61-8), aluminum nitrate nonahydrate (Al(NO₃)₃·9 H₂O, ≥ 98%, CAS: 7784-27-2), and benzene-1,3,5-tricarboxylic acid (BTC, ≥ 99%, CAS: 554-95-0) were purchased from commercial suppliers.

### Synthesis of Fe-Al @BTC MOF

Fe-Al @BTC MOF was synthesized by using a mixture of (2.70 g, 0.01 mol) of iron chloride, (3.75 g, 0.01 mol) of aluminium nitrate, and (2.10 g, 0.01 mol) of 1,3,5-benzenetricarboxylate (BTC) in 10 ml dimethylformamide (DMF). The temperature of the reaction mixture was 120 °C. The reaction was put in the oven for 5 h. The yellow precipitate was formed, filtered, and dried in an oven at 60 °C.

### Instrumentation

X-ray diffraction (XRD) patterns were recorded using a Shimadzu XRD-6000 diffractometer equipped with Cu Kα radiation (λ = 1.5406 Å, 45 kV, 40 mA) to investigate the crystalline structure of the synthesized Fe–Al@BTC MOF. Transmission electron microscopy (TEM) was performed using a 200 kV transmission electron microscope to examine the internal morphology and particle size distribution. Scanning electron microscopy (SEM) was employed to analyze surface morphology, and elemental composition was determined using energy-dispersive X-ray spectroscopy (EDX) attached to the SEM instrument. Ultraviolet–visible (UV–Vis) absorption spectra were obtained using a JASCO V-750 spectrophotometer. Fourier transform infrared (FT-IR) spectra were recorded in the range of 400–4000 cm⁻¹ using a Thermo Fisher Scientific spectrometer with KBr pellets. Raman spectra were collected using a WITec alpha300 R Raman spectrometer. Photoluminescence (PL) measurements were carried out using a He–Cd laser (325 nm) coupled with a HORIBA iHR320 spectrometer. Positron annihilation lifetime spectroscopy (PALS) measurements were conducted using a fast–fast coincidence ORTEC system with a ²²Na source to investigate free-volume characteristics. Electrochemical measurements, including Mott–Schottky analysis and electrochemical impedance spectroscopy (EIS), were performed using a CHI660E electrochemical workstation.

### Structural simulation and powder X-ray diffraction analysis

A reflex module implemented in Materials Studio^27^ was used to derive the structure of crystals from the powder diffraction data. The Reflex module determined with the stimulated PXRD patterns *P212121* space group, was chosen for the primitive models in the initial simulations^28^. X-Cell is applied to identify the crystalline type and the estimated lattice parameters according to the positions of peaks in the powder pattern^29,30^.

### Computational details

Utilizing the Gaussian 09 W program^31^. The theoretical investigation was carried out through( DFT/B3LYP) methods and 6-31G (d, p) basis set level using the Berny method^32,33^. No symmetry constraints were applied during the geometry optimization. The wide-ranging vibrational modes were assigned using the Potential Energy Distribution (PED), determined by the Vibrational Energy Distribution Analysis (VEDA) program^34^.

### Bacterial strain and culture conditions

*Bacillus cereus* is a Gram-positive bacterium that was isolated from drainage water and molecularly identified using 16 S rRNA gene sequencing. B. cereus strain was grown on agar plates containing tryptic soy agar media^35^. These agar plates were re-streaked every seven days and kept at 4 °C until the time of injection.

#### Minimum inhibitory concentration (MIC) of MOF

Single colonies of *Bacillus cereus* strain were introduced into tryptic soy broth, and cultures were maintained in an incubator shaker at 200 rpm, 37 °C for 18 h, and then adjusted to a concentration of 1 × 10^7^ CFU/mL using the 0.5 McFarland standard. 5 µL of Bacterial broth, except for the negative control, was introduced to experimental flasks containing 20 ml of tryptic soy broth media and different concentrations of MOF. The cultures were maintained in an incubator shaker at 200 rpm, 37 °C for 24 h. The initial and final spectrum readings at OD600 were acquired before and after 24 h of flask incubation at 37 °C.

The monitoring was carried out using a fourfold serial dilution of the MOF systems at concentrations ranging from 200 mg/L to 1,000 mg/L. MIC values were defined as the lowest MOF concentration that suppressed observable bacterial growth. Experiments were performed in triplicate. The initial concentration at which no growth occurred was termed MIC^36^.

#### Minimum bactericidal concentration (MBC) of MOF

Tryptic soy agar plates were subcultured from flasks used in the minimum inhibitory concentration assay to obtain the minimum bactericidal concentrations (MBC). After 24 h of incubation at 37 °C, the MBC value was recorded as the lowest concentration of MOF that reduced the viability of the original bacterial inoculum by ≥ 99.9%, as evaluated by visual inspection. The MBC is the lowest concentration supplied, which resulted in bacterium death.

### Molecular docking studies

The molecular docking of MOF was performed by PyRx and visualized by PyMOL. Although all conformational analysis was carried out with the 0.01 Å RMS slope, the energy of the acquired shapes was reduced. Threonine synthase from *Bacillus* subtilis ATCC 6633 with PLP and APPA (**PDB ID**: 6NMX) was used for molecular docking. Ten distributed docking simulations were carried out using standard conditions, with verification based on the E shape and the layout of general parameters.

## Results and discussion

### Chemistry

**Scheme 1:**
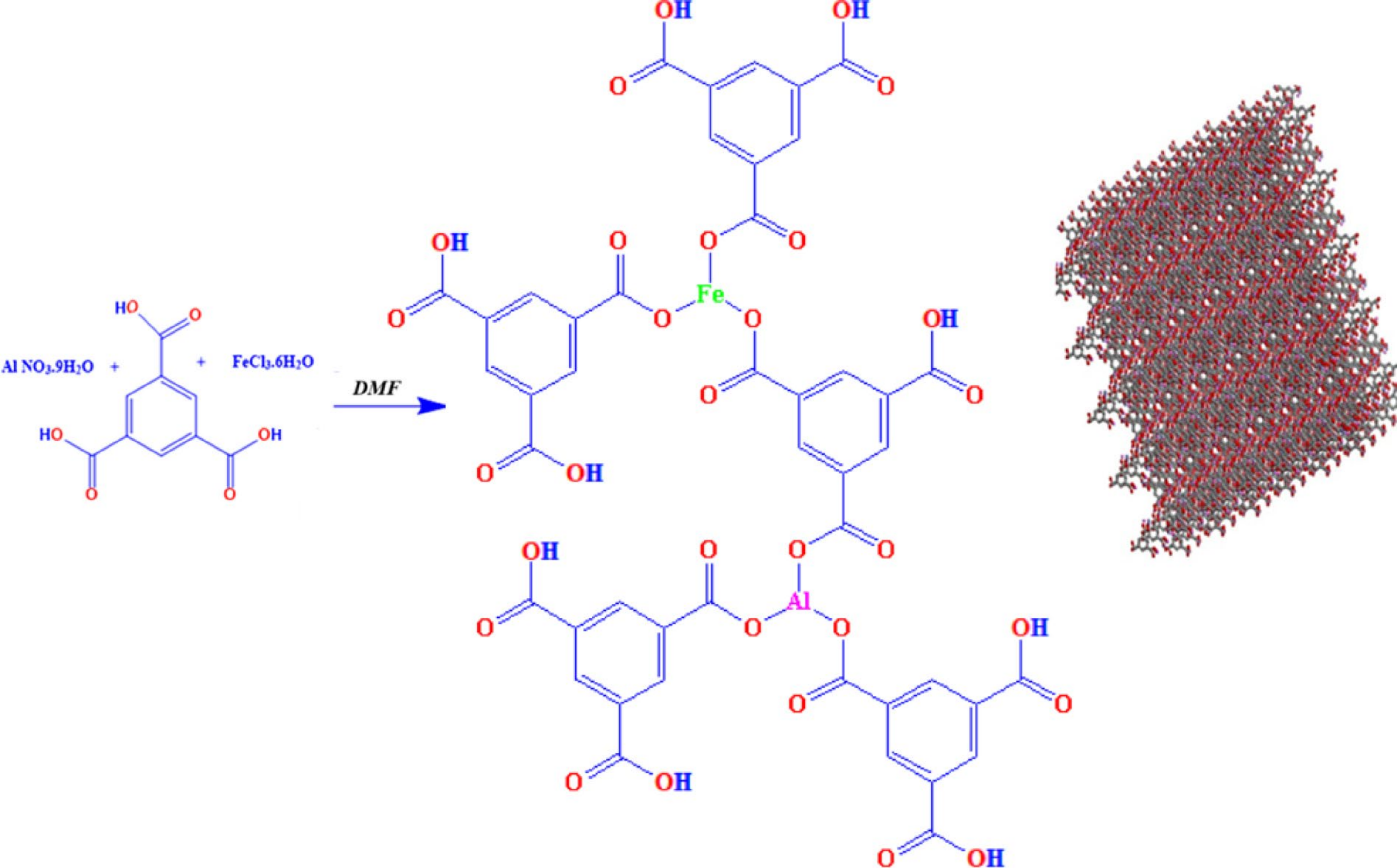
Synthesis route of Fe-Al @BTC metal-organic framework under the hydrothermal method.

Fe-Al @BTC MOF was synthesized under a hydrothermal reaction. A mixture of (0.01 mol) of iron chloride and (0.01 mol) of aluminium nitrate were reacted with (0.01 mol) of 1,3,5-benzenetricarboxylate (BTC) in 10 ml of dimethylformamide (DMF). Fe-Al @BTC MOF was formed in yellow solid powder as expressed in (Scheme 1).

#### Fourier transform infrared (FT-IR) spectral analysis

**Fig. 1:**
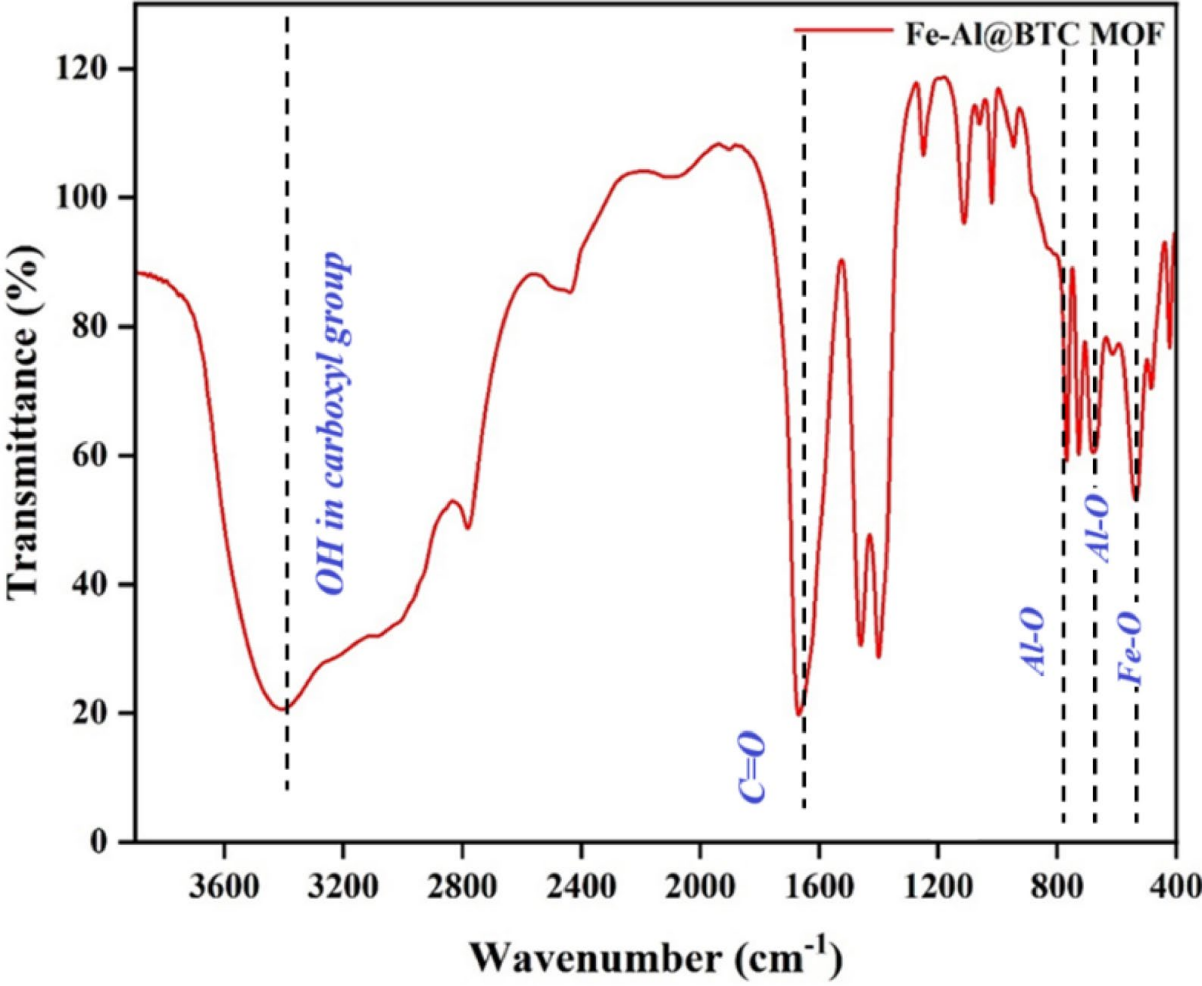
FT-IR spectrum for Fe-Al@BTC MOF.

The FTIR spectra of Fe-AL@BTC, showed a broad band in the region of 3600 –3200 cm^− 1^ referred to O-H stretching vibrations of the carboxyl group (COOH) as shown in (Fig. 1). The symmetric and asymmetric stretching vibrations of the carboxyl group originating from H_3_BTC are shown at 1369 cm^− 1^ and 1621 cm^− 1,^ respectively. The observed findings show that the metal center coordinates successfully with the -COO groups in a bidentate manner. 550 cm^− 1^, the peak is specifically attributed to Fe-O vibrations, while the peaks at 510 cm^− 1^ and 1080 cm^− 1^ correspond to Al-O vibrations.

#### UV-visible analysis

**Fig. 2:**
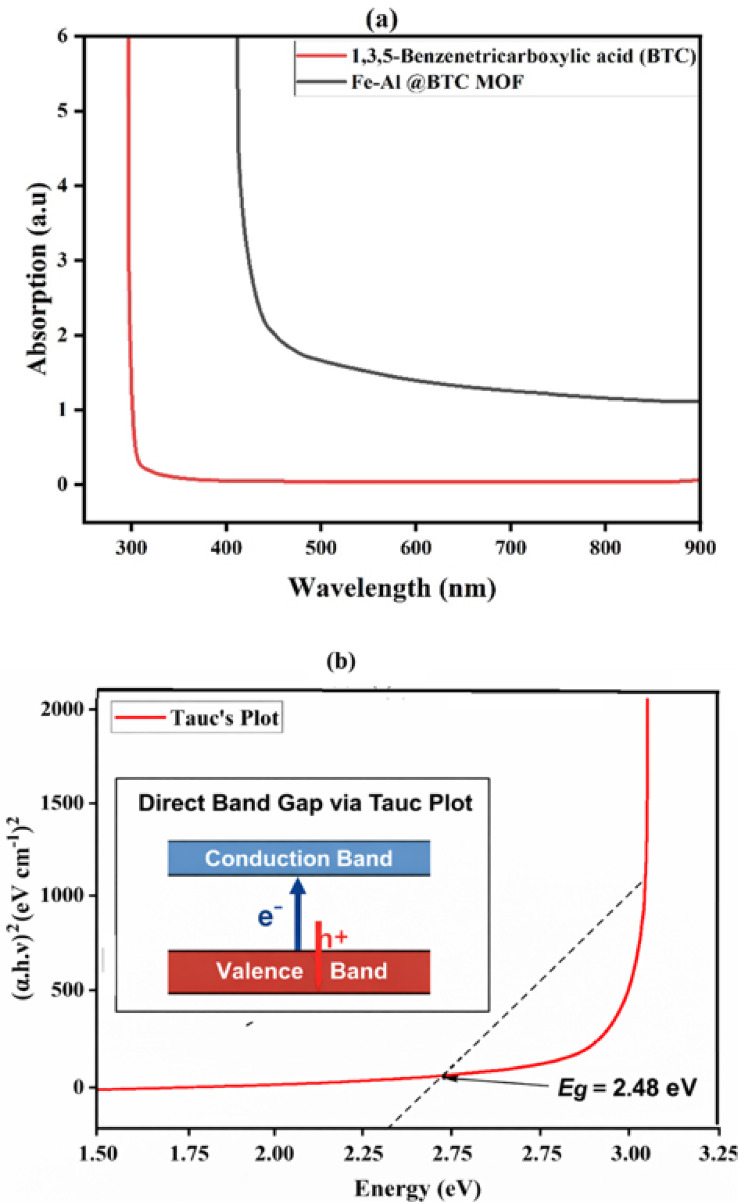
(**a**) UV-V spectrum of Fe-Al@BTC metal-organic framework, (**b**) Tauc’s graph for the band gap measurement.

The optical properties of the synthesized Fe–Al@BTC MOF were investigated using UV–visible absorption spectroscopy. As shown in Fig. 2(a), the absorption spectrum exhibits a broad absorption band extending into the visible region, indicating strong light-harvesting capability arising from metal–ligand interactions within the framework. The red shift observed compared to the BTC ligand confirms the successful coordination between the Fe³⁺/Al³⁺ metal centers and the organic linker. The optical band gap energy of Fe–Al@BTC MOF was determined using the **Tauc relation**:

$$\left( {\alpha h\nu )^{n} = A(h\nu - E_{g} } \right)$$

where $\alpha$is the absorption coefficient, $h\nu$is the photon energy, $A$is a constant, and ${E}_{g}$is the optical band gap. For a direct allowed electronic transition, the exponent $n=2$; therefore, a plot of $(\alpha h\nu )^{2}$versus $h\nu$ was employed.

As illustrated in Fig. 2(b), the linear region of the Tauc plot was extrapolated to intersect the photon energy axis. The intercept of the tangent line with the energy axis yields an optical band gap value of 2.48 eV. The presence of a well-defined linear region confirms that the optical transition in Fe–Al@BTC MOF follows a direct band gap mechanism. This direct band gap behavior facilitates efficient electronic transitions and charge carrier excitation, which is favorable for electrochemical and photoelectrochemical applications.

The obtained band gap value is consistent with the observed optical absorption behavior and supports the enhanced electrochemical performance of the Fe–Al@BTC MOF, particularly in supercapacitor applications where rapid charge transfer is required. The conduction band (CB) and valence band (VB) edge positions of the Fe–Al@BTC MOF were estimated based on the optical band gap obtained from UV–Vis spectroscopy. Using the determined band gap value of 2.48 eV and the flat-band potential analysis, the CB and VB positions were calculated to be at suitable energy levels, enabling efficient charge transfer processes. These band edge positions further support the electrochemical and antibacterial performance of the Fe–Al@BTC MOF.

#### Raman spectroscopy analysis

**Fig. 3:**
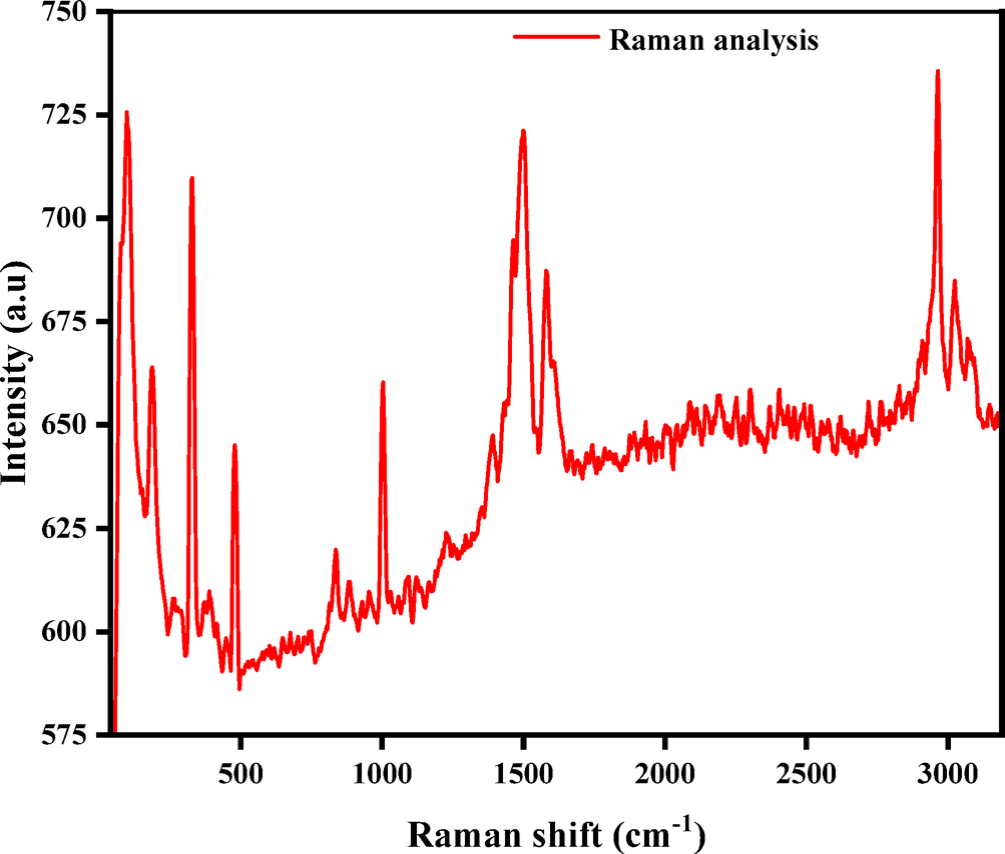
Raman spectra of Fe-Al @BTC MOF sample.

The Raman spectroscopy results prove that the functionalization of Fe and Al metal nodes through BTC linkers gets accomplished which is vital for the retention of stability and aromatic content of the MOF as shown in Fig. 3. The peaks in the spectra suggest that the metal-organic framework synthesized by the solvothermal method using DMF has assimilated enough metal ions and organic linkers into a stable form. In particular, the peak that occurs at 330 cm^− 1^ is due to M-O (Fe-O or Al-O) vibration suggesting that there are metal nodes at the junction of the organic linker^37^. The peaks observed at 801 cm^− 1^ and 930 cm^− 1^ belong to the titers of the C-H bending of the benzene ring of the BTC linker, reaffirming the aromatic framework^38^. At 1003 cm^− 1^, C-O stretching is associated with a carboxylate group, indicating the interaction between the metal ions and the BTC molecule. At 1507 cm^− 1^ and 1580 cm^− 1^ in turn, represent carbon–carbon double bond (C = C) stretching vibrations in carbon-carbon double bonds and are sustained by the aromatic rings in MOF^39^. Lastly, the peaks recorded at 2965 cm^− 1^ and 3023 cm^− 1^ have been associated with C-H stretching modes affirming the occurrence of the benzene ring and organic framework^40^.

#### Powder X-ray diffraction of Fe-Al@ BTC MOF

**Fig. 4:**
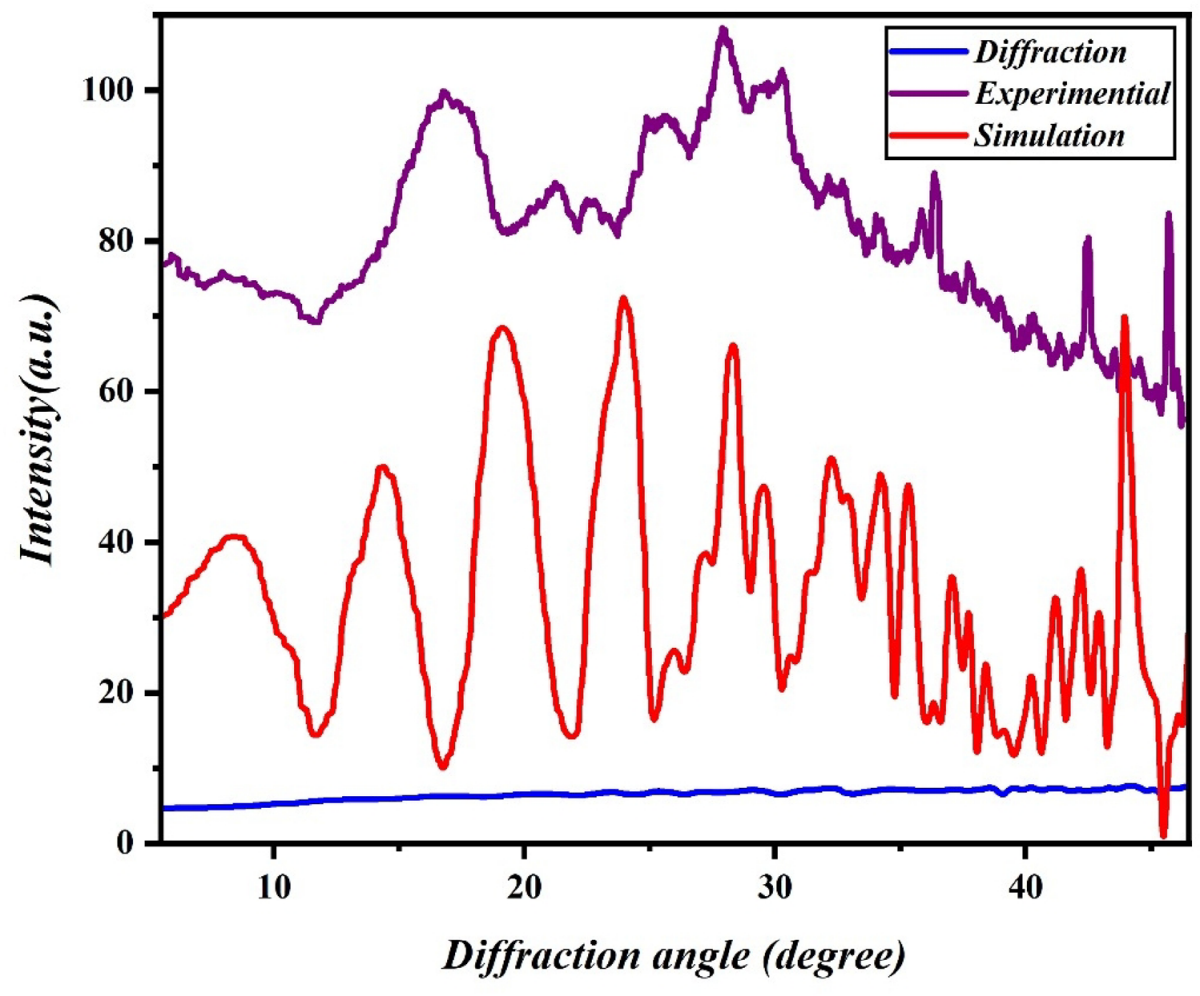
XRD pattern of Fe-Al@ BTC MOF.

The crystalline structure of Fe–Al@BTC MOF was examined by powder X-ray diffraction (PXRD). Characteristic peaks were observed at 2θ = 9.7°, 18.5°, 20°, 23.8°, and 28.3° (Fig. 4), consistent with BTC-based MOFs and confirming the formation of a crystalline framework. The relatively low intensity and broadening of the peaks, particularly the first reflection at 2θ ≈ 9.7°, indicate reduced long-range order, which is commonly seen in nanoscale or porous MOFs. This reduced order reflects the presence of small crystalline domains rather than porosity itself, as PXRD alone cannot directly measure pore structure. The slight broad peak at ~ 18.5° can be attributed to π–π stacking interactions between MOF layers. A decrease in overall peak intensity may suggest a minor partial collapse of the crystalline structure during synthesis^41,42^.

High-resolution TEM (HRTEM) further supports the PXRD observations. Lattice fringes with d-spacing values of 0.34 nm and 0.21 nm correspond to planes inferred from PXRD reflections, confirming the presence of crystalline domains within the MOF. Moreover, the honeycomb-like morphology observed in TEM images aligns with the PXRD and HRTEM results, collectively indicating that Fe–Al@BTC MOF possesses a semi-crystalline, porous structure. Such structural features are favorable for efficient ion diffusion and pseudocapacitive charge storage in supercapacitor applications.

The computational analysis was conducted using P21212 space group with α = β = γ = 90°, and unit cell dimensions of a = 26 Å, b = 18 Å, and c = 24 Å for the Fe-Al @BTC MOF. The unit cell parameters were determined by indexing the PXRD peaks using the X-Cell algorithm implemented in the Reflex module of Materials Studio. The calculated unit cell dimensions were refined by profile fitting, and the agreement between simulated and experimental patterns was evaluated using Rwp and Rp reliability factors. The low values of Rwp (9.1%) and Rp (7.8%) indicate good agreement between the refined model and experimental data.

These parameters closely matched the experimental structure. The R_wp_ and R_p_ values converged to 9.1% and 7.8%, respectively. The crystallite size has been calculated for Fe-Al@ BTC MOF from x-ray line broadening method by Scherer’s equation:

1 $$\boldsymbol{D}=\frac{\boldsymbol{K}\boldsymbol{\lambda}}{{\boldsymbol{\beta}}_{\boldsymbol{h}\boldsymbol{k}\boldsymbol{l}}\mathbf{cos}\boldsymbol{\theta}}$$

where D is the crystallite size, k the shape factor (0.94), $\lambda$ the wavelength of CuKα radiation, $\theta$ the Bragg diffraction angle in radians and ${\beta}_{hkl}$ the peak width at half-maximum intensity in radians. It was found that the average crystal size for Fe-Al@ BTC MOF is 81 nm.

According to Eq. (1), the previous parameters of Fe-Al@BTC MOF were calculated using the solvothermal method, as shown in Table 1, where the X-ray wavelength equals 1.54 Å.

**Table 1:** X-ray structure properties of Fe-Al@ BTC MOF.

Pos. [2θ°.]	FWHM [2θ°.]	Crystallite sizes D (nm)	Dislocation density (δ)
9.63	**0.0787**	**100.94**	**9.81462E-05**
18.70	**0.0787**	**100.94**	**9.81462E-05**
23.83	**0.0985**	**80.66**	**0.000153703**
26.65	**0.0590**	**134.64**	**5.51635E-05**
28.35	**0.2362**	**33.63**	**0.000884191**
31.18	**0.1574**	**50.47**	**0.000392585**
37.78	**0.1574**	**79.44**	**0.000158461**
43.89	**0.1181**	**67.26**	**0.000221048**
		**Average = 80.9975**	**Average = 0.000152425**

#### Transmission electron microscopy (TEM)

**Fig. 5:**
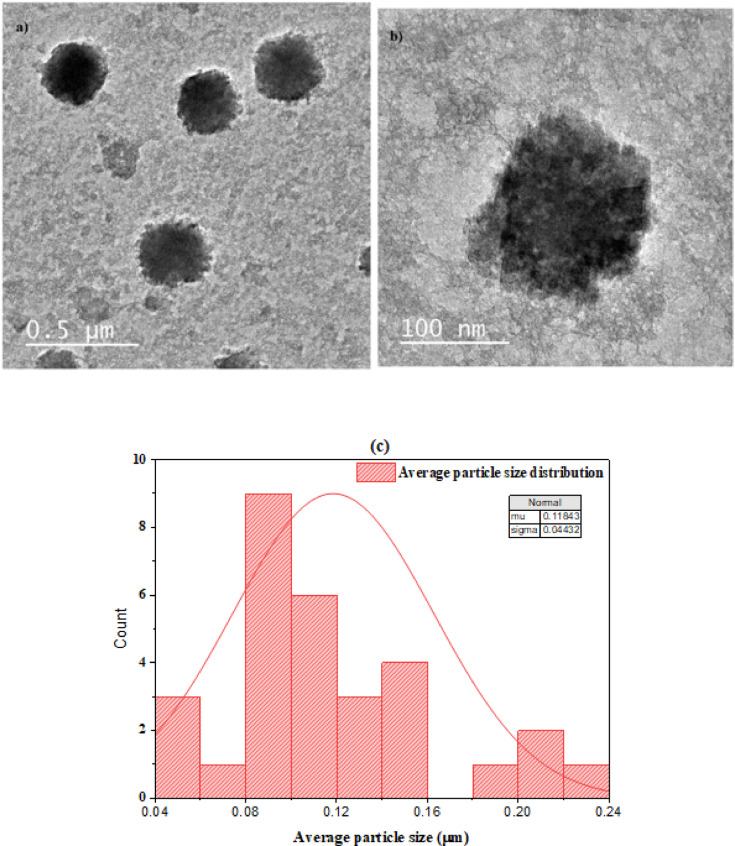
(**a**,**b**) Micrographs of Fe-Al@ BTC MOF taken by conventional TEM (**c**) Average particle size distribution.

The Fe-Al@ BTC MOF’s TEM image, as shown in Fig. 5 (a, b) displays a network of linked grains or particles with a structure like a honeycomb. Higher electron density in the darker areas suggests thicker or denser sections inside the particles. Lower electron density in the lighter regions suggests thinner or less dense material. The image accentuates the complex pattern that particle interactions produce. The average particle size distribution was determined using Image-J software, and the results showed that the average particle size was around 118 nm as shown in Fig. 5(c). The smaller particle size observed in TEM images compared to SEM is attributed to the higher spatial resolution of TEM and the visualization of individual crystallites rather than agglomerates.

#### Scanning electron microscope (SEM)

**Fig. 6:**
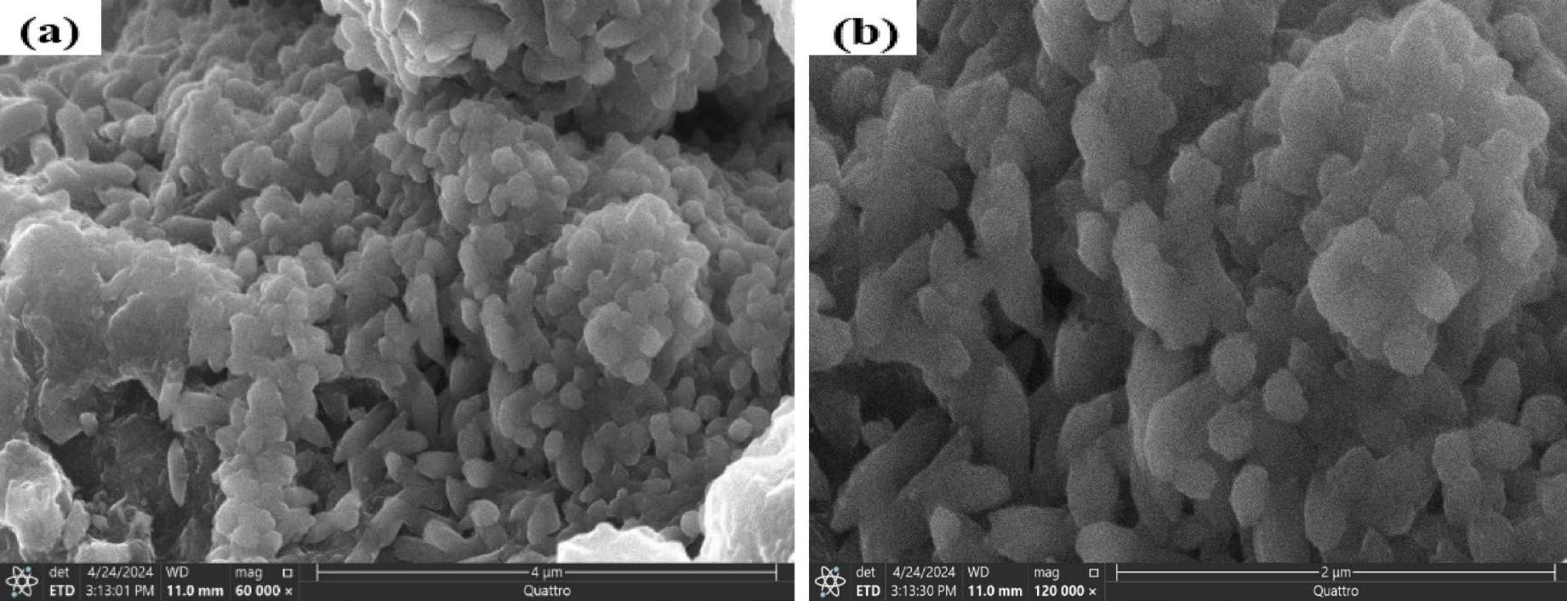
(**a**,**b**) Top- view SEM images of Fe-Al@BTC MOF.

The synthesized Fe-Al@BTC MOF exhibits a porous orthorhombic structure with sharp edges, suggesting high crystallinity and purity as evident in the SEM images shown in Fig. 6(a, b). The morphology also features agglomerated clusters with a cauliflower-like texture, which is characteristic of MOFs formed by agglomerated particles and pores. The grain size of the Fe-Al@BTC led to the formation of ultra-small pores with a diameter of approximately 170 nm, as confirmed by TEM analysis. This pore size remains consistent in the composite sample.

Quantitative analysis of the SEM images was performed using ImageJ software. The Fe–Al@BTC MOF particles exhibit an average particle size of approximately 118 nm, with a size distribution ranging from 90 to 150 nm. This quantitative analysis confirms the relatively uniform morphology of the synthesized material and supports the structural observations discussed above.

**Fig. 7:**
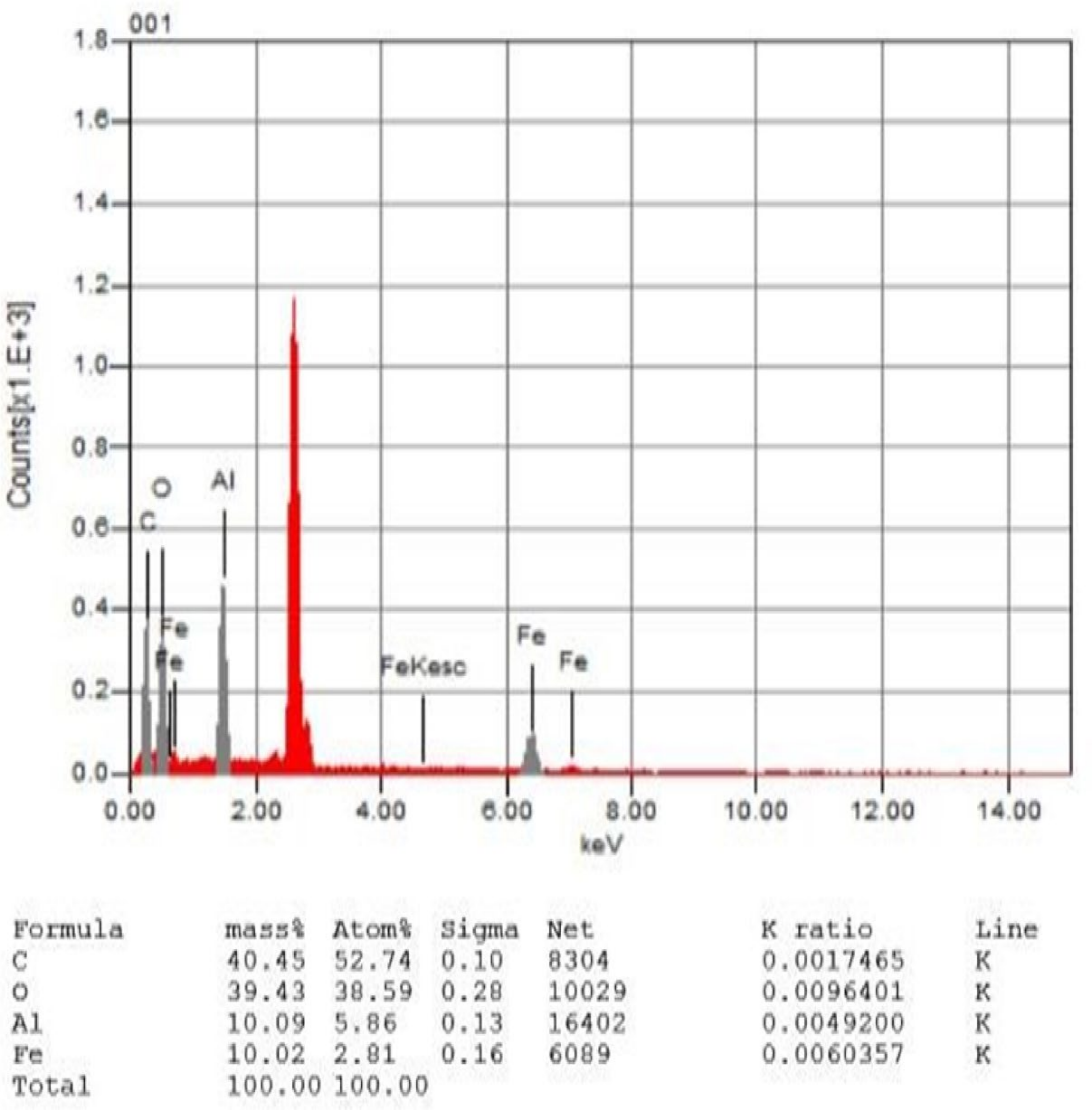
Energy dispersive X-ray (EDX (analysis of Fe-Al@BTC MOF.

The estimated elemental composition of the Fe-Al BTC MOF crystals, determined using SEM-EDX, is presented in Fig. 7. The EDX analysis reveals prominent peaks indicating the presence of aluminum (Al) and iron (Fe) in the sample, synthesized at a solvothermal temperature of 120 °C for 17 h. The empirical formula for the Fe-Al@BTC MOF derived from this study is approximately _C40.45_ O_39.47_ Al_10.09_ Fe_10.02_.

#### Positron annihilation spectroscopy (PAS)

PAS is a valuable technique used to investigate the structure and defects within materials by analyzing the interaction of positrons with electrons in the material PALS provides valuable insights into MOF porosity, we used PALS to determine the amount of the lifetime, intensity, and the free volume that presented in the Fe-Al@ BTC MOF. According to the free-volume model^43^, the lifetime of o-Ps localized within a rigid and spherical potential well with a radius of R_0_, and a free volume with a radius of R, where no electrons are present below this limit, is determined by the following Eqs^44,45^. :

2 $${\boldsymbol{\tau}}_{3}=0.5{\left[1-\left(\frac{\boldsymbol{R}}{{\boldsymbol{R}}_{0}}\right)+\left(\frac{1}{2\boldsymbol{\pi}}\right)\boldsymbol{S}\boldsymbol{i}\boldsymbol{n}\left(\frac{2\boldsymbol{\pi}\boldsymbol{R}}{{\boldsymbol{R}}_{\boldsymbol{O}}}\right)\right]}^{-1}$$

where represents $\partial R = R_{0} - R = 1.656~\hbox{\AA}^{O}$ the empirically fitted electron layer thickness. Using this value of $\partial R$, the radius R of the free volume was computed from Eq. (2), and the average size of the free volume voids V_f_ was assessed as V_f_ = (4/3)π R^3^ (in Å^3^). Samples were analyzed using positron annihilation spectroscopy, a technique that probes the interactions of positrons with matter. The analysis revealed two distinct components in the positron annihilation lifetime spectrum: an intermediate-lived component (τ_1_ = 2.530 ns) and a long-lived component (τ_2_ = 0.41682 ns). These components were associated with different annihilation processes: τ_1_ corresponds to the annihilation of free positrons and para-positronium (p-Ps), whereas τ_2_ is attributed to ortho-positronium (o-Ps) annihilation The intensities of these components were measured as I_1_ = 2.139 and I_2_ = 97.861, respectively, indicating a predominance of o-Ps annihilation in the sample. The fitting variance is 1.7190 and the free volume is 3.9640, which are indicative of the quality of the fit and the free volume within the material, respectively as shown in Table 2.

**Table 2:** Positron parameters of Fe-Al@ BTC MOF.

Symbol	${\boldsymbol{\uptau}}_{1}$	I_1_	${\boldsymbol{\uptau}}_{2}$	I_2_	*R* (Å)	V	${\boldsymbol{\upchi}}^{2}$
Fe-Al @BTC	**2.530 (0.013)**	**2.139 (0.013)**	**0.41682 (0.00062)**	**97.861 (0.013)**	**3.9640**	**149.54**	**1.7190**

#### Photoluminescence analysis

The photoluminescence (PL) spectrum of the Fe–Al@BTC MOF exhibits multiple emission peaks corresponding to distinct electronic transitions within the framework. The electrochemical performance of the Fe–Al@BTC MOF electrode was evaluated using a three-electrode configuration, which allows accurate separation of the working electrode behavior from the counter and reference electrodes. In this system, the Fe–Al@BTC MOF acts as the working electrode, Ag/AgCl serves as the reference electrode to provide a stable potential, and a platinum electrode functions as the counter electrode to complete the circuit.

The observed charge storage behavior originates from a combination of electric double-layer capacitance (EDLC) and pseudocapacitive (faradaic) processes. The EDLC contribution arises from the adsorption of electrolyte ions at the electrode–electrolyte interface, while the pseudocapacitive behavior is associated with reversible redox reactions involving Fe²⁺/Fe³⁺ redox couples within the MOF structure. This synergistic mechanism enhances charge storage capacity, rate capability, and electrochemical stability.

**Fig. 8:**
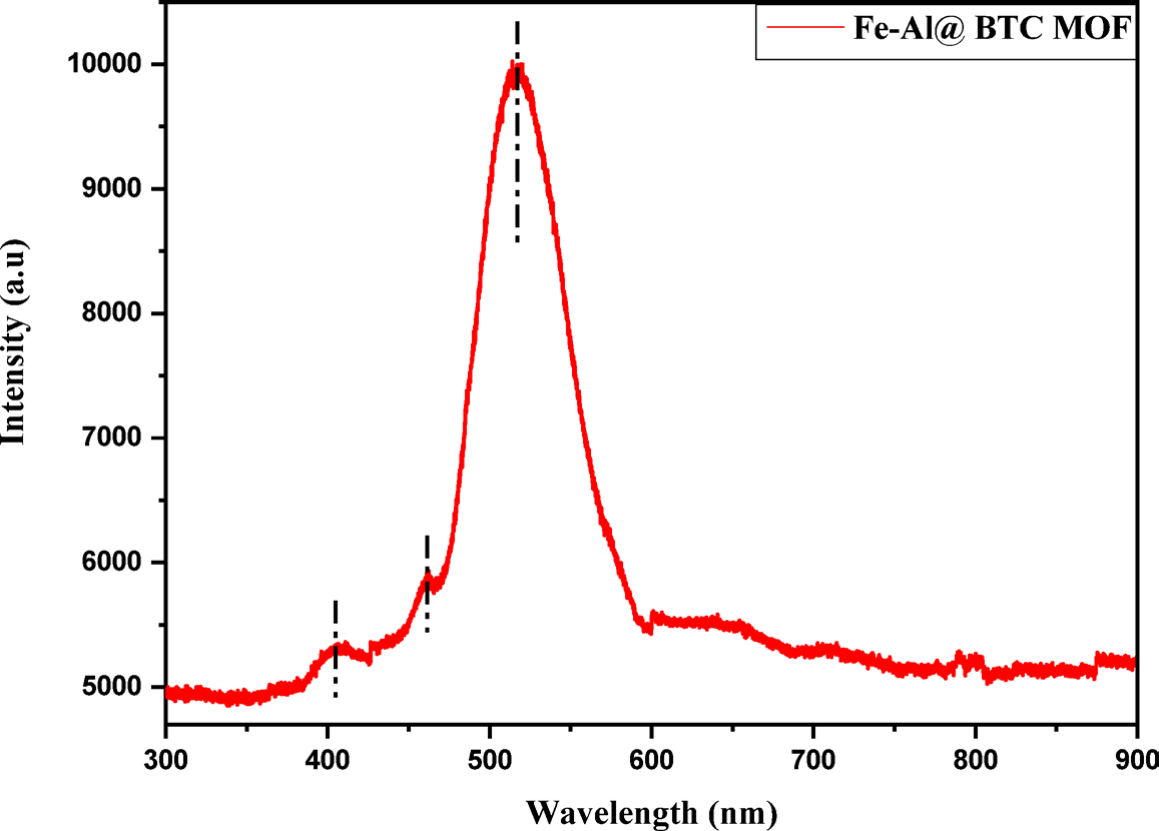
Photoluminescence (PL) analysis for Fe-Al@ BTC MOF.

The emission band centered at 405 nm (3.06 eV), located in the near-ultraviolet region, can be attributed to high-energy electronic transitions, primarily arising from ligand-to-metal charge transfer (LMCT) or metal-to-ligand charge transfer (MLCT) processes involving the Fe³⁺/Al³⁺ metal centers and the π-conjugated benzene-1,3,5-tricarboxylate (BTC) linker. These transitions indicate efficient electronic coupling between the organic ligand and metal nodes^46^. The second emission peak observed at 461 nm (2.68 eV), in the blue region of the visible spectrum, is associated with electronic transitions involving the excited states of the metal centers and the organic linker. This emission can be assigned to a combination of charge transfer transitions and d–d electronic transitions, particularly influenced by the Fe ions. The specific coordination environment of Fe within the MOF lattice facilitates such transitions by modifying the crystal field and electronic density around the metal centers, thereby enhancing radiative recombination processes^47^. A pronounced and intense emission peak appears at 516 nm (2.40 eV), corresponding to the green region of the visible spectrum. This strong emission is attributed to defect-related states and intra-ligand transitions within the MOF framework. Structural imperfections, such as oxygen vacancies, metal coordination defects, and lattice distortions, introduce localized energy states within the bandgap, which act as radiative recombination centers. Additionally, the strong metal–ligand interactions in the Fe–Al@BTC framework enhance these defect-mediated transitions, resulting in increased emission intensity. The presence of this dominant green emission suggests a high density of defect states and efficient charge recombination pathways, which are consistent with the observed optical and electronic properties of the MOF, as illustrated in Fig. 8.

#### Mott Schottky analysis

**Fig. 9:**
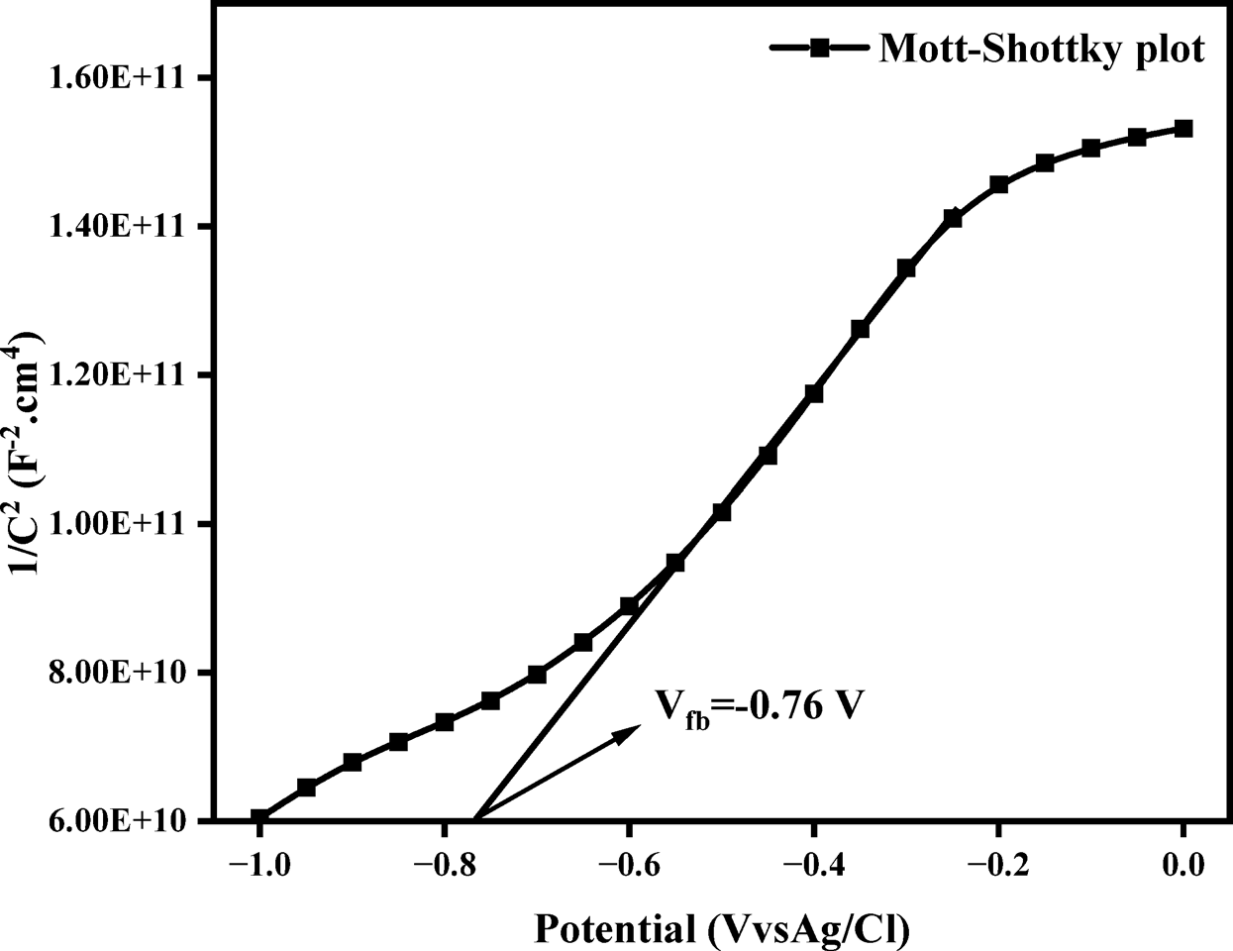
Mott–Schottky plot for Fe-Al @BTC MOF.

Mott–Schottky analysis was employed to determine the flat-band potential and carrier density of the Fe–Al@BTC MOFdue to its suitability for semiconducting and porous electrode materials. This method provides direct insight into charge carrier behavior at the electrode–electrolyte interface, offering reliable estimation of band edge positions when combined with optical band gap data. To assess the electrical properties of the fabricated materials, the Mott–Schottky technique was employed to calculate the carrier density and determine the conductivity type (n-type or p-type). The graph of 1/C^2^ is plotted against V by using 1 M KOH as the supporting electrolyte, the Mott-Schottky curve is measured^48^. The Mott–Schottky relation for the fabricated Fe–Al@BTC MOFis displayed in Fig. 9 and was measured at a frequency of 1 kHz with an amplitude of 5 mV. In the cathodic direction, the capacitance was measured in the potential range of -1 to 1 V. The following Mott-Schottky Eq. (3) determines the space charge capacitance of the Schottky junction (C), the built-in potential or flat-band potential (V_fb_), the carrier concentration (N_D_), and additional parameters, such as the electron charge (e), temperature (T), the applied potential (v), Boltzmann constant (K_B_), the dielectric constant of material (e) the dielectric permittivity of the vacuum (e_0_), and the area in contact with the junction (A)^49–51^.

3 $$\frac{1}{{\boldsymbol{C}}^{2}}=\frac{2}{-\boldsymbol{\upepsilon}{\boldsymbol{\upepsilon}}_{0}\mathbf{e}{\mathbf{A}}^{2}{\mathbf{N}}_{\boldsymbol{D}}}(\mathbf{V}-{\mathbf{V}}_{\mathbf{f}\mathbf{b}}-\frac{\mathbf{k}\mathbf{T}}{\mathbf{e}})$$

The transfer in band edge positions at the semiconductor-fermi level electrolyte interface is the cause of the built-in voltage fluctuation. The difference in Fermi levels between the semiconductor and electrolyte at the interface serves as a gauge of the level of the barrier at the point of contact, which is indicated by V_fb_^52^. The Schottky barrier potential at the electrode/electrolyte interface exhibits a linear response, from which the flat band potential (V_fb_) may be determined by stretching toward the x-axis, where N_D_ values are estimated from the following Eq. (4)^53^.

4 $${\boldsymbol{N}}_{\boldsymbol{D}}=\frac{2}{-\boldsymbol{\upepsilon}{\boldsymbol{\upepsilon}}_{0}\mathbf{e}{\mathbf{A}}^{2}\mathbf{S}}$$

where S characterizes the slope of Mott–Schottky graph. From the graph the calculated value of V_fb_ is -0.76 V and the slope is 1.06393E11, so it was found the donor carrier density is 1.3E20 cm^− 3^ and it is confirmed the type of conductivity material is n-type.

#### Electrochemical impedance spectroscopy measurements

All electrochemical measurements, including cyclic voltammetry, galvanostatic charge–discharge, and electrochemical impedance spectroscopy, were performed independently in this work using freshly prepared Fe–Al@BTC MOF electrodes. The electrochemical performance claims in this study are based on three-electrode measurements, which are widely used for fundamental material evaluation. Although the obtained energy density values appear relatively high, they represent intrinsic material performance rather than full device-level metrics.

The study investigated the interfacial properties between electrodes and electrolytes using electrochemical impedance spectroscopy (EIS) measurements. We use for measurement Fe–Al@BTC MOF with DMF solution in 0.5 M KOH solutions. Also examined at 7 ml Fe–Al@BTC MOF with DMF solution in 3 ml KOH of 1.0 V of potential and 10^4^ Hz of frequency^54,55^.

**Fig. 10:**
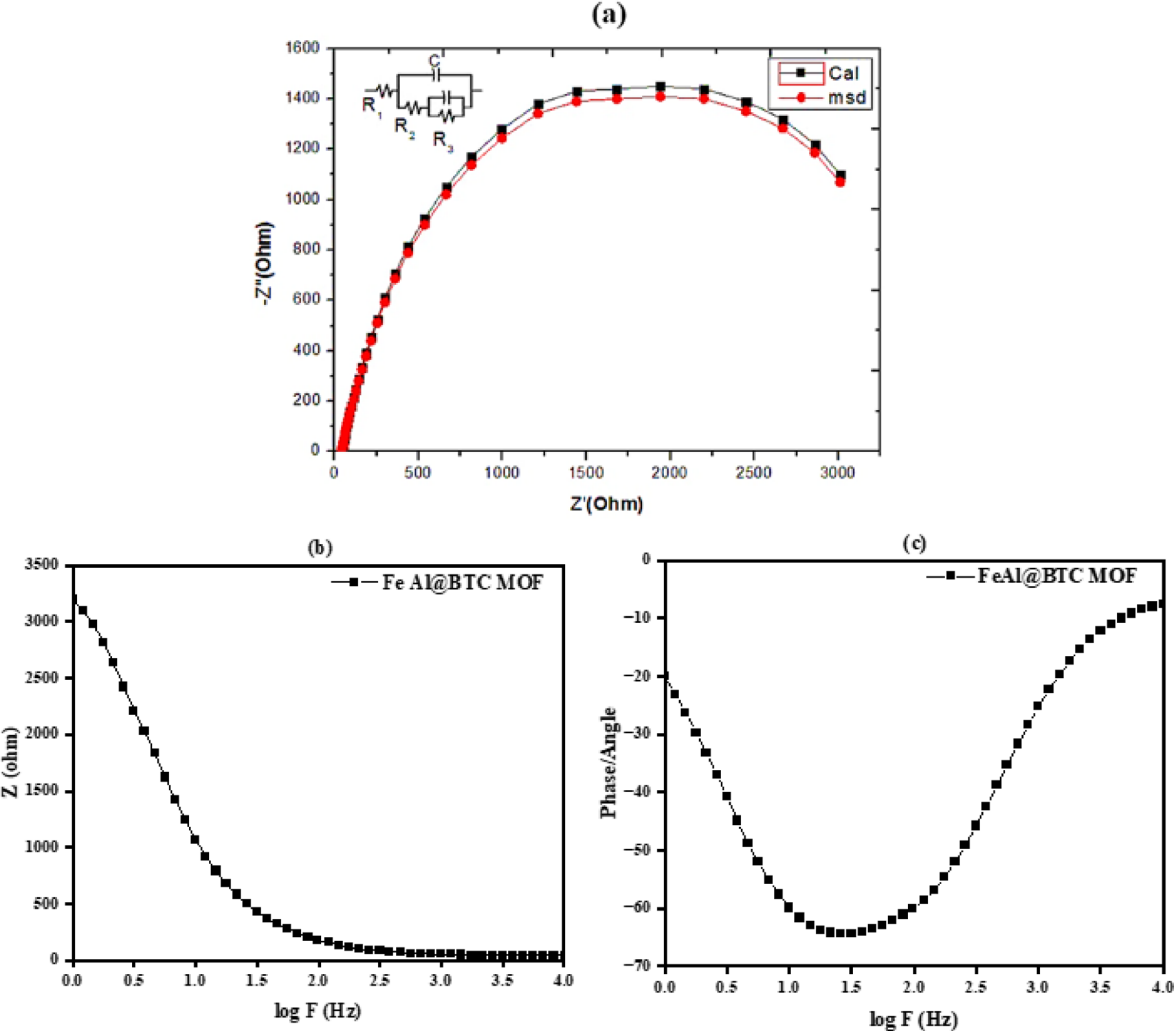
EIS analysis of Fe-Al@BTC MOF (**a**) Nyquist plots, (**b**) and (**c**) shows (∣Z∣) vs.log F and phase angle vs.log F for the Fe-Al@BTC MOF, respectively.

As shown in Fig. 10(a) the Nyquist plots were used to evaluate the actual impedance (Z′) values versus negative imaginary values (Z′′) curves of a sample^55–57^. The Nyquist plot arc, a measure of charge transportation resistance, was used to analyze the results. An equivalent circuit R (C (R (C (R))) was used to match the obtained spectra, which included the constant phase element (CEP: C), R_1_ represents the resistance of solution, R_2_ refers to the resistance of charge transfer, and R_3_ represents the adsorption resistance^54,58^. The plots were fitted to a semicircle using the Simpkin program. The Nyquist plot in the image shows a semi-circular arc, indicating the presence of a charge transfer resistance and double-layer capacitance at the electrode/electrolyte interface. The equivalent circuit model with resistors and capacitors suggests that the Fe–Al@BTC MOF exhibit good electrochemical properties, with the measured data closely matching the calculated data^59–61^. This implies stable and efficient material for potential applications in corrosion resistance or battery technology^62–65^. The phase angle diagrams in Fig. 10(c) further validate the presence of semi-circular arcs, which help in understanding the charge transfer mechanism at the Fe–Al@BTC MOF electrolyte interface. The diameter of these semi-circles typically corresponds to the charge transfer resistance (R_ct_). To explore this behaviour more thoroughly, bode plots in Fig. 10(b, c) were used. Figure 10(b) displays the impedance magnitude (∣Z∣) and phase angle as a function of frequency (log(f)), offering insights into the system’s capacitive and resistive characteristics. In the high-frequency range, the impedance magnitude is lower, indicating a decrease in solution resistance (R_s_), while at lower frequencies, the impedance magnitude increases due to more efficient ion diffusion. Furthermore, in the mid-frequency range, the impedance magnitude decreases, signifying a reduction in charge transfer resistance (R_ct_). In Fig. 10(c), the Bode plot shows how the impedance magnitude and phase angle vary with frequency. At high frequencies, the phase angle is close to 0°, reflecting the solution resistance (R_s_).

In the mid-frequency range, the phase angle represents both the charge transfer resistance (R_ct_) and the double-layer capacitance (C_dl_) at the Fe-Al @BTC MOF-electrolyte interface^66^.

5 $${\boldsymbol{C}}_{\boldsymbol{d}\boldsymbol{l}}=\frac{1}{2\boldsymbol{\pi}{\boldsymbol{f}}_{\boldsymbol{m}\boldsymbol{a}\boldsymbol{x}}{\boldsymbol{R}}_{\boldsymbol{c}\boldsymbol{t}}}$$

This EIS analysis highlights the efficient electrochemical behaviour of the Fe–Al@BTC MOF in energy storage applications.

The deviation observed in the kinetic fitting of the Fe–Al@BTC MOF system suggests that the electrochemical process may not be governed solely by ideal kinetic models. Additional factors such as ion adsorption, diffusion-controlled processes, and surface heterogeneity of the MOF electrode may contribute to the overall electrochemical behavior. These effects can lead to discrepancies between experimental data and simplified kinetic models, as commonly reported for porous MOF-based electrodes.

A Ragone plot was constructed to compare the energy density and power density of the Fe–Al@BTC MOF electrode with previously reported supercapacitor materials. The Fe–Al@BTC MOF exhibits competitive energy and power density values, highlighting its potential for high-performance energy storage applications.

## Application for Fe-Al@BTC MOF

### Supercapacitor studies

**Fig. 11:**
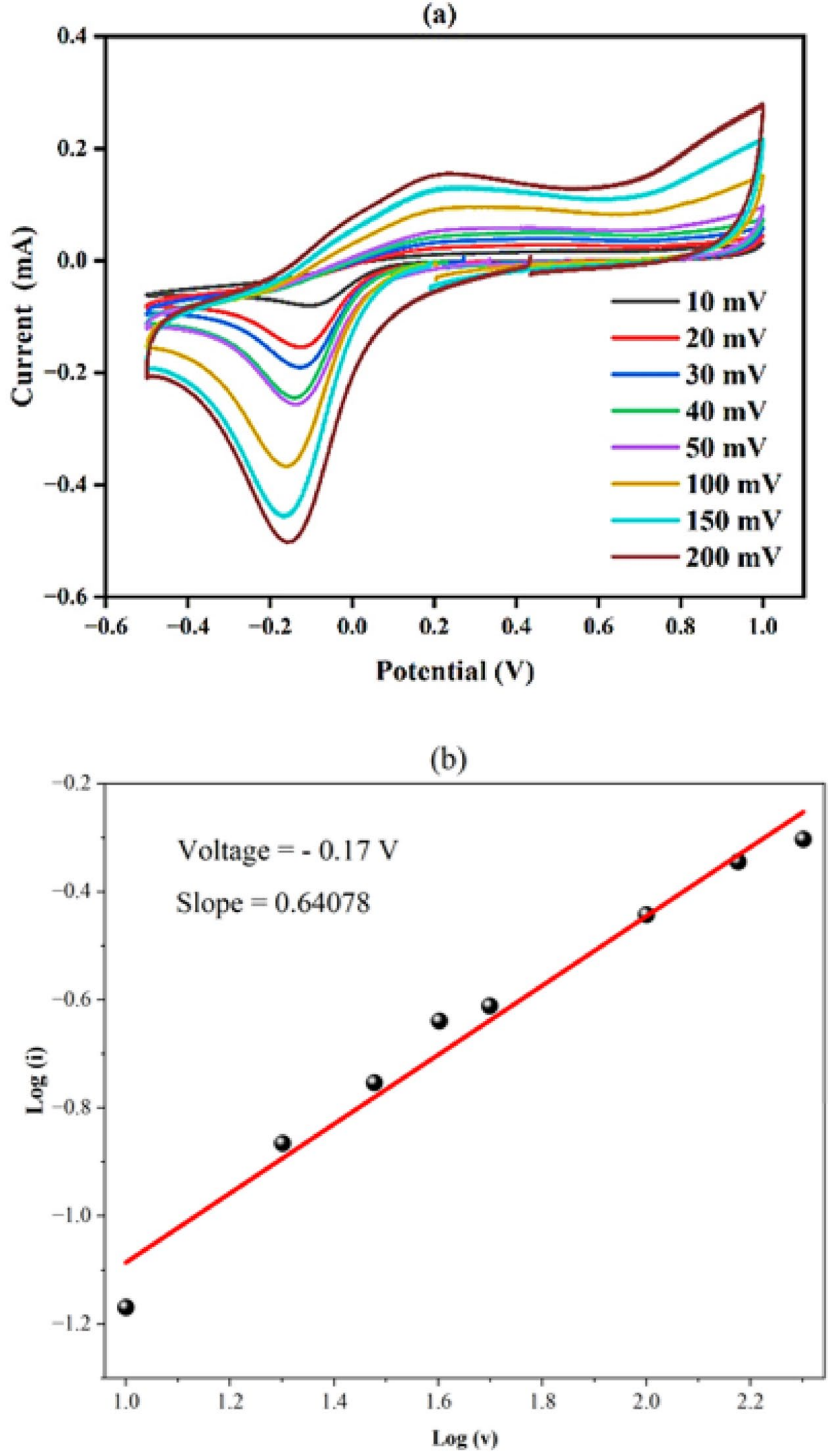
(**a**) CV curves, and (**b**) The relationship between log of scan rate and log of electrical current from cyclic voltammetry data at -0.17 V.

The electrochemical performance of the Fe-Al@BTC MOF electrode for supercapacitor applications was studied using a three-electrode system in 0.5 M KOH aqueous electrolyte. The working electrode is fabricated as follow: a small amount of active material is dissolved within deionised water with the addition of small amount of PVP as a binder to the solution then apply ultrasonic for 15 min for achieve well dispersed solution. Adding few drops of solution onto FTO substrate placed at hot plate at moderate temperature via drop casting technique. The low temperature decreases the surface tension of water to produce well distributed film after the evaporation of water. The mass of active material is determined by measuring the mass of substrate before and after deposition which was found approximately 3 mg. The cyclic voltammetry (CV) analysis, illustrated in Fig. 11(a), provides insight into the electrochemical behaviour of this material. The CV curve depicts the performance of the Fe-Al@BTC MOF over a potential range of -0.5 to 1 V at various scan rates (10, 20, 30, 40, 50, 100. 150, and 200 mV/s). The experimental setup consisted of an Ag/AgCl reference electrode, a platinum counter electrode, and a working electrode composed of 3 mg of Fe-Al@BTC MOF deposited on FTO substrate as a current collector forming the working electrode^55^.

At slower scan rates, such as 10 mV/s, the CV curve exhibits the lowest enclosed area and the smallest current peak while at the higher scan rate the enclosed and current peak become larger. At low scan rates, capacitive current is smaller → less current throughout the cycle → smaller loop area. At higher scan rates, capacitive current increases → broader CV curves → larger enclosed area. The presence of two distinct peaks corresponds to reversible redox reactions within the framework, with the anodic and cathodic peaks representing oxidation and reduction processes of electroactive species, likely involving Fe²⁺/Fe³⁺ ions. Fe²⁺/Fe³⁺ redox activity is well established and fully plausible within typical CV potential windows. Al³⁺ is electrochemically inactive under these conditions because Al³⁺ has a very negative reduction potential (Al³⁺/Al ≈ − 1.66 V vs. SHE). It does not undergo reversible redox transitions in aqueous or mild electrochemical windows because the potential window in this experiment is limited from 1 to -0.5 V and Therefore, the observed redox behavior of the Fe–Al@BTC MOF can be primarily attributed to the reversible Fe²⁺/Fe³⁺ redox couple. In contrast, Al³⁺ is electrochemically inactive within the applied potential window and does not participate in redox processes. The role of Al³⁺ is therefore structural, contributing to framework stability and modulation of the electronic environment around Fe active sites rather than directly participating in charge transfer^67^. During the forward scan, the oxidation of Fe²⁺ to Fe³⁺ occurs as OH⁻ ions from the electrolyte intercalate into the Fe-Al@BTC MOF structure, indicating a faradaic process. This faradaic behaviour is responsible for the redox reactions that store energy through electron transfer and ion intercalation, contributing to the overall charge storage^68^. In contrast, during the reverse scan, deintercalation of OH⁻ ions occur, reducing Fe³⁺ back to Fe²⁺. Additionally, at higher potentials, the system exhibits typical pseudocapacitive charging behaviour.

The CV curves at different scan rates further reveal both diffusion-controlled and capacitive controlled processes. The relationship between peak current (*i*) and scan rate (*v*) in cyclic voltammetry follows:

$$i=a{v}^{b}$$

where i represent the electrical current due to reduction or oxidation process, v is the scan rate and b-value determine the dominant mechanism diffusive or capacitive controlled.

Taking logarithms:

$$\mathrm{l}\mathrm{o}\mathrm{g}i=\mathrm{l}\mathrm{o}\mathrm{g}a+b\mathrm{l}\mathrm{o}\mathrm{g}v$$

So, by plotting log(i) vs. log(v), the slope = b.

If b ≈ 0.5 the diffusion-controlled process is dominant storage mechanism (typical battery-type Faradaic redox reactions), while if b ≈ 1.0 the capacitive-controlled process is the dominant storage mechanism (surface-controlled processes: EDLC or pseudocapacitance) while when 0.5 < b < 1.0 mixed behavior is the dominant mechanism^69^. As shown in Fig. 11(b), the slope equal to b-value which is around 0.64078 demonstrating the mixed behavior of capacitive and diffusion-controlled contributions.

As the scan rate increases, the current density also increases, reflecting faster electron transfer kinetics. However, the absence of sharp redox peaks suggests that broad, multi-step redox processes are taking place, likely due to the multiple redox-active sites within the Fe-Al@BTC framework. This faradaic behaviour highlights the strong electrochemical stability and reversibility of Fe-Al@BTC MOF, making it an excellent candidate for energy storage devices like supercapacitors^70^. Although cyclic voltammetry provides valuable insight into charge storage behavior, the specific capacitance values obtained from CV are considered quantitative^71^. The specific capacitance values discussed in this study are primarily derived from CV curves, which provide reliable and reproducible information on charge storage efficiency, coulombic efficiency, and electrochemical reversibility of the Fe–Al@BTC MOF electrode during oxidation and reduction processes. Thus, the Fe-Al@BTC MOF electrode demonstrates a mixture of faradaic and capacitive behaviours, contributing to its high charge storage capacity and stability. The symmetrical arrangement of the peaks and uniform capacitive region in the CV curves indicate excellent reversibility and potential for long-term use in energy storage. The charge storage characteristics can be quantified through further analysis of the CV data, reinforcing the material’s suitability for supercapacitor applications^72–75^.

Based on the optical band gap value of 2.48 eV, the conduction band and valence band positions of the Fe–Al@BTC MOF are estimated to be approximately − 0.7 eV and + 1.78 eV, respectively. The following Equation determines the specific capacitance of Fe-Al@BTC MOF^76^.

6 $$\mathbf{C}\mathbf{p}=\frac{\int\mathbf{I}\left(\mathbf{V}\right)\mathbf{d}\mathbf{v}}{2\mathbf{m}\mathbf{s}\boldsymbol{\Delta}\mathbf{V}}$$

where Cp is the fabricated material’s specific capacitance in Farad per gram, m represents the mass of the active material in grams, I(V) is the current in mA, and the integral area under the CV curve is denoted as ∫ I (V) dV. The potential window (ΔV) is in Volts, the scan rate (s) is in millivolts per second. As the scan rate increases, Fe–Al@BTC MOF shows a decrease in specific capacitance, despite an increase in the area under the curve, which is attributed to the ion exchange process. Ions have sufficient time to penetrate the pores when the scan rate is slow, resulting in a large charge and specific capacitance^77^. At a scan rate of 10 mV/s, the specific capacitance from cyclic voltammetry is 339.24 F/g, using Eq. (6). The increased capacitive behavior of Fe–Al@BTC MOF was due to well-organized micropores and mesopores that facilitate electrolyte diffusion into the electrode sample, enabling maximal redox reaction extent^78^.

#### Optimization of MOF

**Fig. 12:**
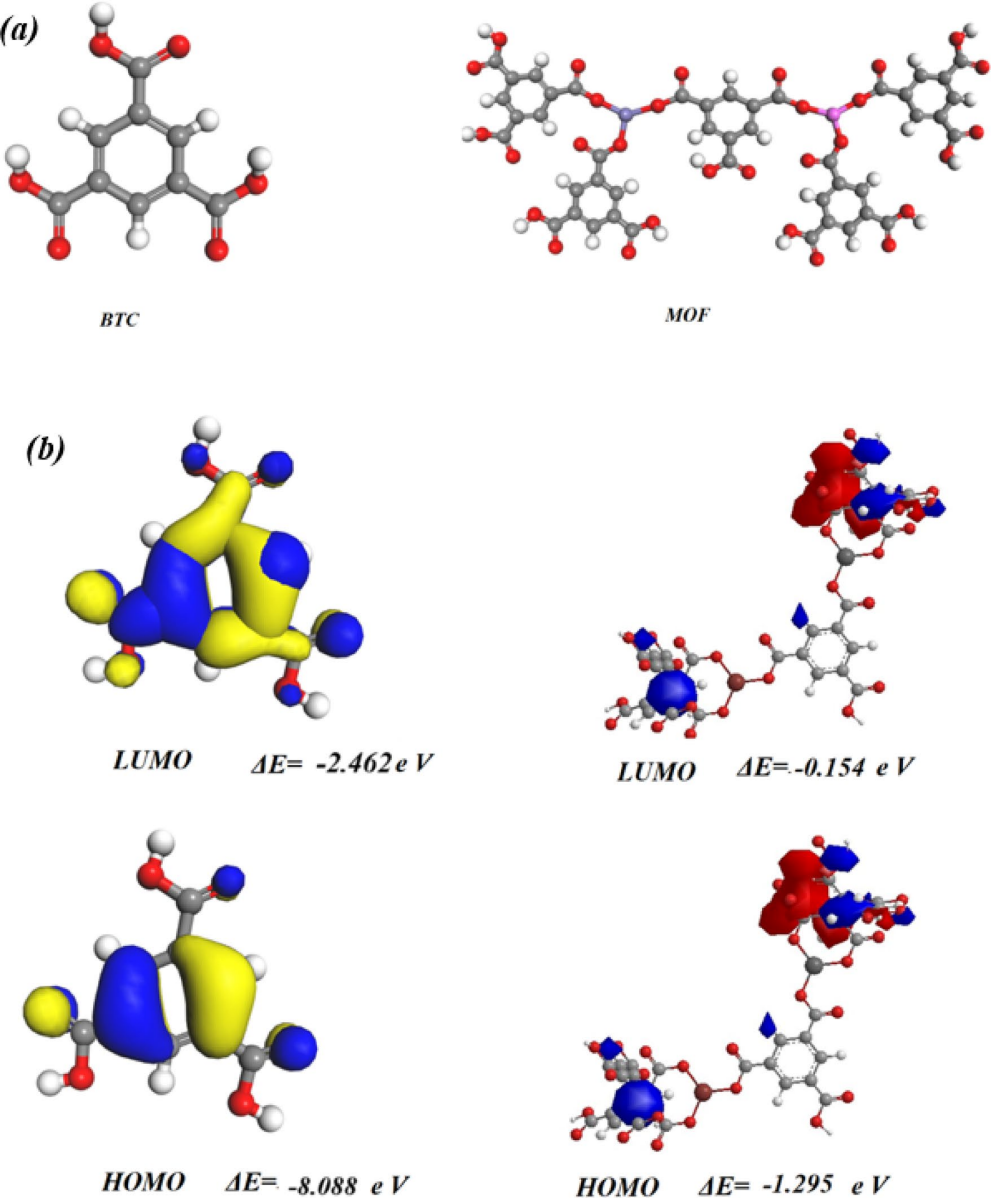
(**a**) Optimized molecular structure of BTC and MOF; (**b**) HOMO-LUMO of optimized structure.

The optimized molecular structure of BTC and MOF was utilized through DFT/ B3LYP 6-31G (d, p) using Gaussian 09^31^ as displayed in Fig. 12, Also, the physical characteristics used in the optimization of molecular structures of MOF from the Eqs^3–9^. concerning (χ)electronegativities, (σ) absolute softness, (η) absolute hardness (ω), (Pi) chemical potential, global electrophilicity, and (S) global softness^79,80^ which were scheduled in Table 5 through a basis set DFT/ B3LYP 6-31G)^81–84^ and showed compounds and reactants were not planar and they showed the ability to form more layers.

7 $$\varDelta\boldsymbol{E}={\boldsymbol{E}}_{\boldsymbol{L}\boldsymbol{U}\boldsymbol{M}\boldsymbol{O}}-{\boldsymbol{E}}_{\boldsymbol{H}\boldsymbol{O}\boldsymbol{M}\boldsymbol{O}}$$

8 $$\mathbf{X}=\frac{-({\boldsymbol{E}}_{\boldsymbol{H}\boldsymbol{O}\boldsymbol{M}\boldsymbol{O}}+{\boldsymbol{E}}_{\boldsymbol{L}\boldsymbol{U}\boldsymbol{M}\boldsymbol{O}})}{2}$$

9 $$\boldsymbol{\upeta}=\frac{({\boldsymbol{E}}_{\boldsymbol{L}\boldsymbol{U}\boldsymbol{M}\boldsymbol{O}}-{\boldsymbol{E}}_{\boldsymbol{H}\boldsymbol{O}\boldsymbol{M}\boldsymbol{O}})}{2}$$

10 $$\sigma =1/\eta$$

11 $$Pi= - \c{X\;\;}$$

12 $$S=1/2\eta$$

13 $$w=P{i^2}/2$$

14 $$DN{\text{ }}\hbox{max} = - {\text{ }}Pi/\eta$$

From Table 3; Fig. 12, it was observed that MOF displayed more stability than BTC due to the lower energy of MOF. Moreover, (χ) absolute electronegativity, which describes the affinity of the atom to interact with a mutual pair of electrons, the BTC showed high electronegativity of C = O with (5.275 eV), which interacts with the Al^+ 3^, Fe^+ 3^ to form the MOF (with 1.449 e V)^85^, (σ) chemical softness designates the ability of an atom or a group of atoms to accept electrons. This showed that the MOF (0. 0.876 eV, ~ 12,8 Kcal/mol) has a higher value than BTC. Demonstrating its ability to take atoms from the inside and capture metals or molecules, while chemical hardness η(eV) indicates the extent of the resistance to the change in the electron cloud density of the system, the MOF showed the least chemical hardness (with 1.141 e V) and cannot change the electron cloud due to the metal complex^86^. Pi: the chemical potential of MOF was (-1.449 e V), which gives it the ability to react and absorb more energy in different temperature ranges^87^. The ω indicated the electrophilic character and electron flow between donor and acceptor^88^ so, MOF showed a higher electrophilic character to absorb electrons (with 5.498 eV)^89^.

HOMO refers to the ionization potential, while the LUMO reproduces the electron affinity. The gap energy between the HOMO and LUMO orbitals indicates the stability and reactivity of the structure. A compound with a large band gap will have high stability and low reactivity, such as BTC ΔE = 5.626 e V. The HOMO of BTC interacts with LUMO of Al^+ 3^ and Fe^+ 3^. LUMO of BTC interacts with LUMO of Al ^+3^and Fe^+ 3^ to produce MOF with a minimum band gap energy of ΔE = 1.141 e V and this energy showed the stability of MOF to give more biological evaluation and higher reactivity^33,90^.

**Table 3:** Ground-state energies of BTC and MOF utilizing DFT/ B3LYP 6-31G and their physical parameters:

DFT/ B3LYP 6-31G		
*Physical descriptors*	*BTC*	*MOF*
*E*_*T*_ *(au)*	-844.03	-5486.8
*E*_*HOMO*_ *(eV)*	-8.088	-1.295
*E*_*LOMO*_ *(e V)*	-2.462	-0.154
*Eg (eV)*	5.626	1.141
*χ (eV)*	5.275	1.449
*η(eV)*	2.813	1.141
*σ(eV)*	0.240	0.876
*P*_*i*_ *(eV)*	-5.508	1.4876
*S(eV)*	-263.217	0.224
*ω(eV)*	4.945	5.498
*ΔN*_*max*_	1.95	1.269

### Antibacterial activity

**Fig. 13:**
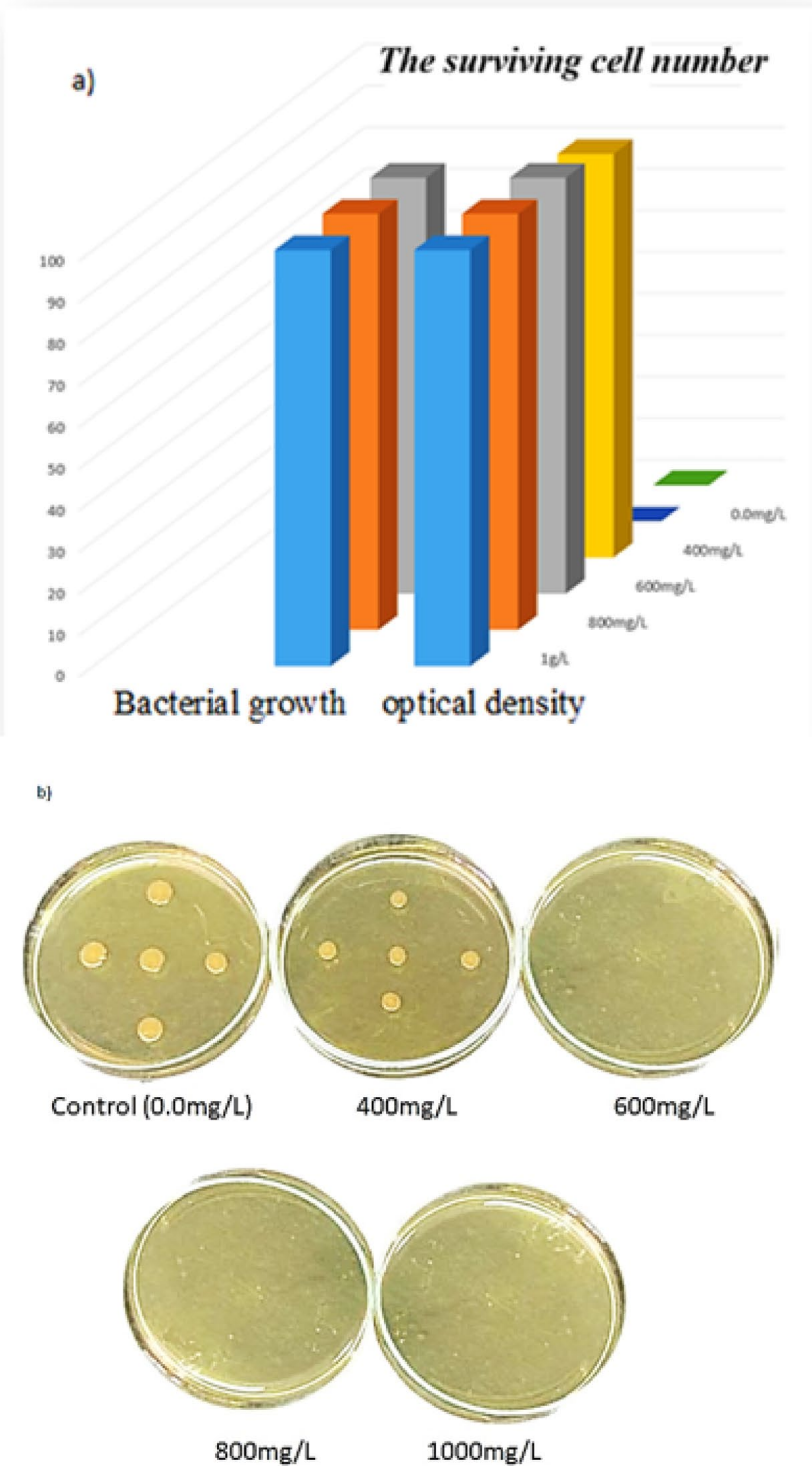
(**a**) The values of surviving cell number of Fe-Al@ BTC MOF by optical density and bacterial growth on LB broth containing different concentrations, (**b**) Image of inhibition zone of Fe-Al@ BTC MOF at different concentrations.

The antibacterial activity of the Fe–Al@BTC MOF was evaluated against Gram-positive bacteria (*Bacillus subtilis*) using the spread-plate method. A control group containing bacterial culture without the MOF was used for comparison to assess the intrinsic antibacterial effect of the synthesized material. The antibacterial efficiency was quantified by measuring the zone of inhibition (ZOI) and calculating the percentage inhibition, providing a quantitative assessment of bacterial growth suppression^91^. Fe–Al@BTC MOF has a high antibacterial impact on the single strain of bacterial cells studied, *Bacillus* bacterial cells. Figure 13a, b shows the antibacterial activity of Fe–Al@BTC MOF at various concentrations on a bacterial culture using optical density (OD) measurements and the cut plug method. The results showed significant activity against *Bacillus* bacteria cells. Fe–Al@BTC MOF showed promising inhibition of bacterial growth by 97% at a concentration of 400 while 100% at a concentration of 600 mg \L by OD measurements. In the case of the cut plug method, Fe–Al@BTC MOF inhibited the bacterial growth by 50% at a concentration of 400 mg \L while 100% at a concentration of 600 mg \L. It was noted that the high efficiency of MOF is due to the following reasons:

1. Electrostatic interactions between the Carbonyl group (C = O) can polarize carbon to bear a positive charge while O bears a negative charge.
2. The Al and Fe metals can coordinate with gram-positive bacteria

Hydrophobic and hydrogen bonding are also likely to have a role in the adsorption of these microorganisms. Cytoplasmic proteins precipitate and coagulate with greater adsorption concentrations. Moreover, a greater increase in bacterial death increases the MOF sensitivity.

Although detailed mechanistic antibacterial experiments were not conducted, the antibacterial activity of the Fe–Al@BTC MOF can be explained through several synergistic mechanisms. The release of Fe³⁺ ions from the MOF framework can interact electrostatically with negatively charged bacterial cell membranes, leading to membrane destabilization and increased permeability. Additionally, defect-rich structures within the MOF, as evidenced by photoluminescence analysis, may facilitate oxidative stress generation, further contributing to bacterial cell damage^92^.

The strong metal–ligand interactions and high surface area of the Fe–Al@BTC MOF enhance contact with bacterial cells, promoting effective antibacterial performance. These mechanisms are consistent with previous reports on MOF-based antibacterial materials and are further supported by molecular docking analysis^26^(Table 4).

**Table 4:** Inhibition zone diameters of Zn-Co@BTC against *Bacillus* bacterial cells.

MOF Materials	Concentration (mg/l)	Inhibition %	OD_600_
Zn-Co@BTC	**0**	**0**	**1.8**
	**200**	**50**	**0.9**
	**400**	**97**	**0.02**
	**600**	**100**	**0**
	**800**	**100**	**0**
	**1000**	**100**	**0**

### Molecular docking studies

**Fig. 14:**
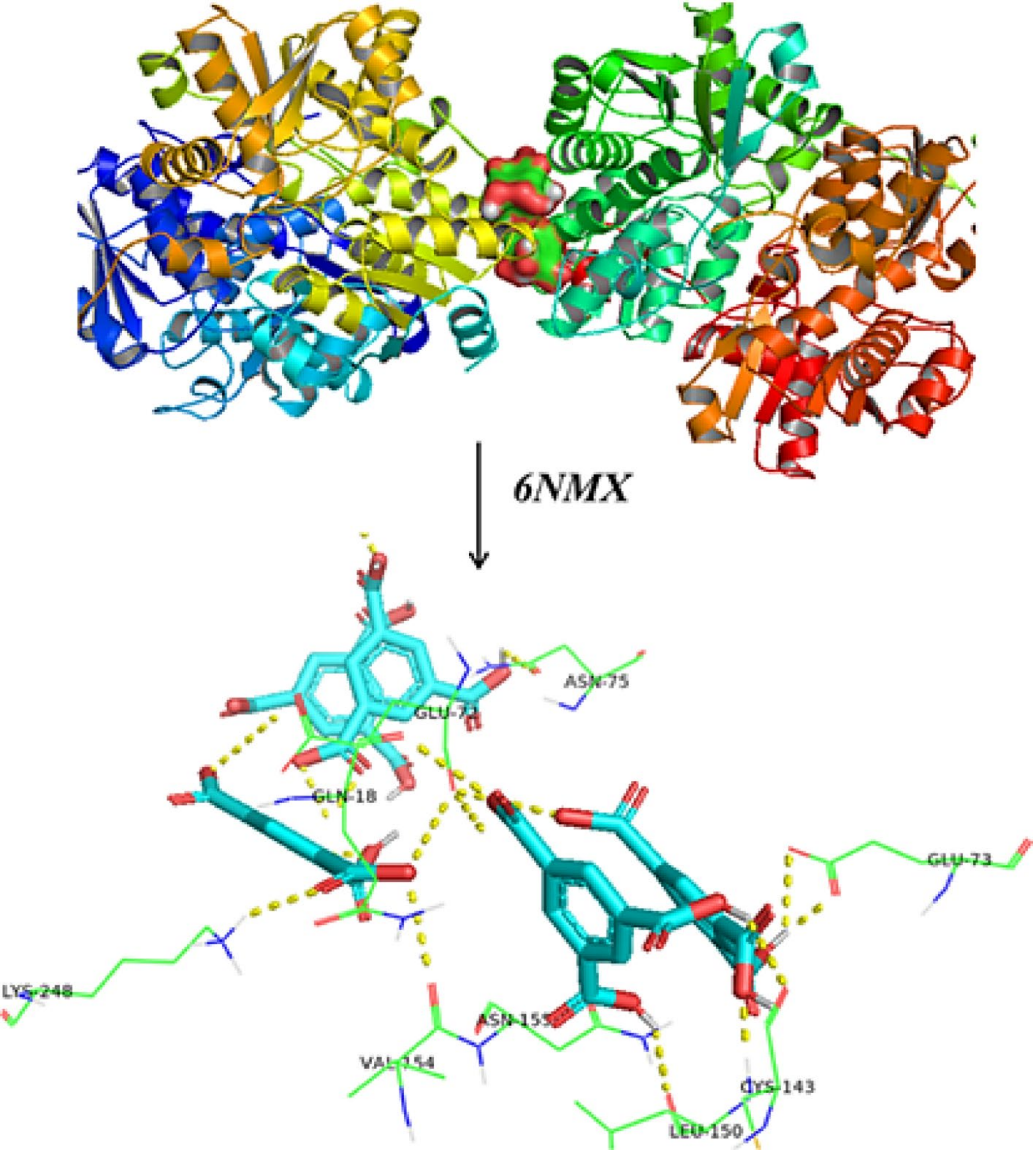
Binding modes of MOF with (**a**) *Bacillus* subtilis (PDB: 6NMX).

The experimental investigation of Fe-Al@BTC MOF’s antibacterial biological activity was supported by molecular docking studies, conducted using the PyRx program, which confirmed consistency between the computational and experimental results^93^. The docking of MOF with threonine synthase from *Bacillus* subtilis ATCC 6633 with PLP and APPA (PDB ID: 6NMX) was used for molecular docking as displayed in Table 5; Fig. 14.

**Table 5:** The molecular docking approaches of MOF with different proteins.

Bacillus subtilis (PDB: 6NMX)			
Fe-Al@ BTC	Energy affinity (kcal/mol)	Distance(Å)	Amino acids
	-12.8 kcal/mol	3.4, 2.5,1.8Å	Glu 72, Glu 73, Glu 18, leu150, lys 195, lys 248, Gys 143, Asn 155, Asn 75, Val 154

The binding energy was observed to be strong between Fe-Al@ BTC and PDBID: 6NMX with − 12.8 kcal/mol, shortage bond length 3.4, 2.5,1.8Å and different amino acids Glu 72, Glu 73, Glu 18, leu150, lys 195, lys 248, Gys 143, Asn 155, Asn 75, Val 154.

**Fig. 15:**
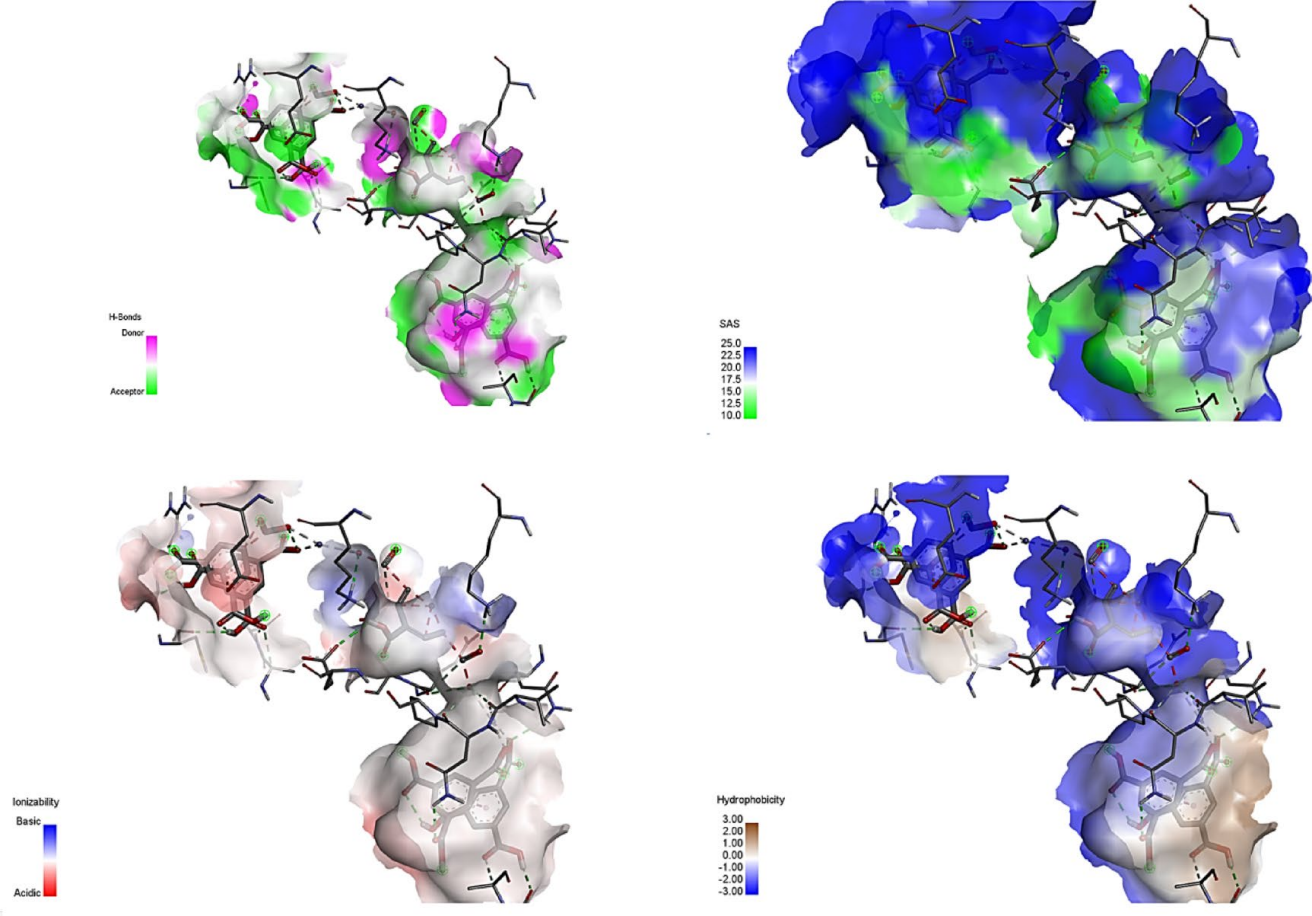
Different binding modes of MOF with *Bacillus* subtilis (PDB: 6NMX).

The antibacterial mechanism of MOF may have occurred through hydrogen bonds, electrostatic interactions, hydrophobic, Vander Waals, and receptor-ligand interactions. These interactions can destroy the membrane of the bacteria and disrupt its cellular components. Hence, it causes the bacteria to die as shown in Fig. 15.

## Conclusion

In conclusion, Fe–Al@BTC MOF was synthesized by the solvothermal method. It showed high performance as a supercapacitor and antibacterial activity against *Bacillus* cells. Different characterization techniques were applied to confirm the structural, optical, morphological, electrical, and photoelectrochemical properties of MOF. Fe–Al@BTC MOF was found to be is n-type semiconductor with a flat band potential of 0.75 V and carrier density of 1.3 × 10^20^ cm^− 3^. The NISTQUEST plot showed a semicircular arc representing the presence of charge transfer resistance and double-layer capacitance at the electrode/electrolyte interface, indicating that the Fe-Al@BTC MOFs exhibit good electrochemical properties This indicated the stability and efficiency of Fe–Al@BTC MOF for potential applications in corrosion resistance and battery technology. The cyclic voltammetry (CV) measurements at different scan rates (10, 20, 30, 40, 50, 100, 150, and 200 mV/s) showed that Fe–Al@BTC MOF has higher performance for supercapacitor application as the capacitive performance of 339.24 F/g was reordered for a slower scan rate of 10 mV/s. Fe-Al @BTC MOF showed significant activity against *Bacillus* bacteria cells where inhibition of 100% at a concentration of 600 mg\L was observed. The antibacterial activity of MOF was supported by molecular docking to understand the interaction with the *Bacillus* subtilis ATCC 6633 protein and its reactivity. Our outcomes indicate that Fe-Al @BTC MOF is a talented contender for supercapacitor and antibacterial applications. Future studies will focus on investigating the influence of synthesis parameters such as reaction temperature, reaction time, and precursor concentration on the structural, electrochemical, and antibacterial properties of the Fe–Al@BTC MOF to further optimize its performance.

## Data Availability

All data generated or analyzed during this study are included in this Article.
